# The Role of Nutritional Factors in Asthma: Challenges and Opportunities for Epidemiological Research

**DOI:** 10.3390/ijerph18063013

**Published:** 2021-03-15

**Authors:** Annabelle Bédard, Zhen Li, Wassila Ait-hadad, Carlos A. Camargo, Bénédicte Leynaert, Christophe Pison, Orianne Dumas, Raphaëlle Varraso

**Affiliations:** 1 Université Paris-Saclay, UVSQ, University Paris-Sud, Inserm, Équipe d’Épidémiologie Respiratoire Intégrative, CESP, 94807 Villejuif, France; wassila.ait-hadad@inserm.fr (W.A.-h.); benedicte.leynaert@inserm.fr (B.L.); orianne.dumas@inserm.fr (O.D.); raphaelle.varraso@inserm.fr (R.V.); 2 Clinical Research Centre, Shanghai First Maternity and Infant Hospital, Tongji University School of Medicine, Shanghai 200092, China; zhen_li@tongji.edu.cn; 3 Department of Emergency Medicine, Massachusetts General Hospital, Harvard Medical School, Boston, MA 02114, USA; ccamargo@partners.org; 4 Service Hospitalier Universitaire Pneumologie, Pôle Thorax et Vaisseaux, CHU Grenoble Alpes, Laboratoire de Bioénergétique Fondamentale et Appliquée, Inserm 1055, Université Grenoble Alpes, 38400 Grenoble, France; cpison@chu-grenoble.fr

**Keywords:** asthma, asthma control, diet, physical activity, body composition, nutritional factors

## Abstract

The prevalence of asthma has nearly doubled over the last decades. Twentieth century changes in environmental and lifestyle factors, including changes in dietary habits, physical activity and the obesity epidemic, have been suggested to play a role in the increase of asthma prevalence and uncontrolled asthma worldwide. A large body of evidence has suggested that obesity is a likely risk factor for asthma, but mechanisms are still unclear. Regarding diet and physical activity, the literature remains inconclusive. Although the investigation of nutritional factors as a whole (i.e., the “diet, physical activity and body composition” triad) is highly relevant in terms of understanding underlying mechanisms, as well as designing effective public health interventions, their combined effects across the life course has not received a lot of attention. In this review, we discuss the state of the art regarding the role of nutritional factors in asthma, for each window of exposure. We focus on the methodological and conceptual challenges encountered in the investigation of the complex time-dependent interrelations between nutritional factors and asthma and its control, and their interaction with other determinants of asthma. Lastly, we provide guidance on how to address these challenges, as well as suggestions for future research.

## 1. Introduction

Asthma is a chronic inflammatory disease of the airways, affecting around 330 million individuals worldwide. Out of 359 diseases, asthma has been classified as the 30th and 32nd cause of disability-adjusted life years (DALYs) in females and males, respectively [1]. It is the most common chronic disease in children [2]. The prevalence of asthma has nearly doubled over the last decades, especially in Westernized countries and developing countries with rapid urbanization [3]. This cannot be explained by genetics alone and it has been hypothesized that this increase is a consequence of changing environmental and/or lifestyle factors [4]. Asthma control, which reflects the disease activity over a short period of time, is suboptimal in more than 50% of adult patients in Europe [5], which has major consequences on health status, quality of life and economic burden [6]. In addition to primary prevention strategies, secondary public health interventions are therefore needed to control existing disease through early detection and appropriate treatment or to reduce the occurrence of exacerbations and the establishment of additional chronic conditions [7].

As recently underlined in *The Lancet* [8], given the immense societal and individual burden of asthma, there is an urgent need to further develop novel strategies to limit or even eradicate the disease. In this context, investigating the role of modifiable lifestyle factors (such as diet, physical activity and, consequently, body composition) is key for the primary and secondary prevention of this common disease. Twentieth century changes in dietary habits (less fruits/vegetables, more ready-to-eat meals), physical activity (less) and the obesity epidemic have been suggested to play a role in the increase of asthma prevalence and uncontrolled asthma worldwide [9]. A large body of evidence has suggested that obesity is a likely risk factor for asthma [10] but mechanisms are still unclear. It has been proposed that obese asthma represents a distinct phenotype of asthma, with one of its main characteristics being poor asthma control [11]. Regarding diet and physical activity, the literature remains inconclusive. Indeed, investigators examining nutritional factors (i.e., diet, physical activity and body composition) in asthma and its control face several challenges. Many definitions and approaches have been used in observational studies for the assessment of asthma and its control, as well as nutritional factors, which makes comparison of study findings a challenge [9]. Furthermore, the role of nutritional factors in asthma may vary throughout life and it is important to consider different windows of exposure. The investigation of the complex interrelations between nutritional factors and respiratory outcomes thus raises major methodological and conceptual challenges in terms of the interpretation and comparison of results, which might explain why definitive findings are scarce.

In this review, we discuss the issue of the assessment of asthma, asthma control and nutritional factors in epidemiological studies, and we provide a non-exhaustive review of the state of the art regarding the role of diet, physical activity and body composition in asthma and its control. Although we give a general overview of the current state of the literature on the role of nutritional factors in asthma for each window of exposure (antenatal period, childhood and adulthood), we focus more specifically on the importance of considering nutritional factors as a whole in this literature, and of addressing the methodological and conceptual challenges raised by the complex time-dependent interrelations between nutritional factors and asthma and its control, and their interaction with genetic, social, environmental and other lifestyle determinants of asthma. Lastly, we provide guidance on how to address these challenges, as well as suggestions for future research.

**Figure 1:**
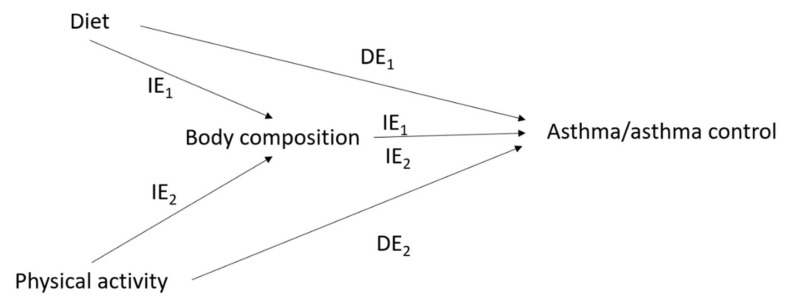
Issue of mediation* in the interrelations between nutritional factors and asthma/asthma control. IE_1_ = indirect effect of diet on asthma/asthma control mediated by body composition; IE_2_ = indirect effect of physical activity on asthma/asthma control mediated by body composition; DE_1_ = direct effect of diet on asthma/asthma control; DE_2_ = direct effect of physical activity on asthma/asthma control; * For simplicity, the time-dependent nature of the interrelations between nutritional factors and asthma, and their covariates, is not represented in this figure (see Figure 2).

In this review, when referring to nutritional factors, we focused on the “diet, physical activity and body composition” triad (Figure 1), and when referring to diet, we focused on food/nutrient intake rather than specific dietary practices such as breastfeeding, infant feeding formula and processed/sterilized food consumption.

**Glossary of terms**: (source: Oxford Languages dictionary)
**Nutrition**: Science that interprets the nutrients and other substances in food in relation to maintenance, growth, reproduction, health and disease of an organism. It includes ingestion, absorption, assimilation, biosynthesis, catabolism and excretion.
**Diet**: In nutrition, it is the sum of food consumed by a person.
**Food**: Any nutritious substance that people or animals eat or drink or that plants absorb in order to maintain life and growth.
**Exercise**: Any bodily activity that enhances or maintains physical fitness and overall health and wellness.
**Physical activity**: Any voluntary bodily movement produced by skeletal muscles that requires energy expenditure. It includes exercise and incidental activity integrated into daily activity.
**Body composition**: In physical fitness, it is used to describe the percentages of fat, bone, water and muscle in human bodies.
**Obesity**: Medical condition in which excess body fat has accumulated to an extent that it may have a negative effect on health.
**Asthma**: Long-term inflammatory disease of the airways of the lungs, characterized by variable and recurring symptoms, reversible airflow obstruction, and easily triggered bronchospasms.

### 1.1. Assessment of Asthma and Asthma Control in Epidemiological Studies 

Asthma is a complex and heterogeneous disease that involves multiple phenotypes or endotypes and windows of expression [12]. Asthma incidence during childhood is greater in boys than in girls, whereas after puberty, asthma incidence is greater in women than in men and remains higher throughout the reproductive years [13]. Early-onset asthma is more often allergic as compared to late-onset asthma. Asthma in the elderly is characterized by more frequent irreversible airway obstruction and accelerated lung function decline due to airway remodeling and sometimes co-existing chronic obstructive pulmonary disease (COPD) as the so-called asthma–COPD overlap (ACO), which was first introduced by the Dutch hypothesis in 1961 [14,15]. The clinical expression of asthma and its control varies over time, both from a long-term perspective (e.g., asthmatic patients may experience periods of remission, sometimes followed by a relapse of symptoms [16]) and from a short-term perspective (e.g., asthma control fluctuates, which can partly be explained by exposure to asthma “triggers” such as tobacco smoke, pets, dust, mites, season, etc.).

Given its phenotypic heterogeneity, the assessment of asthma is challenging. In most epidemiological studies, childhood asthma is assessed using standardized questionnaires on respiratory symptoms, and wheezing patterns during childhood have been proposed as phenotypes of interest [17]. Standardized questionnaires have also been widely used in epidemiological studies to assess asthma incidence or prevalence in adulthood, most of the time using a dichotomous definition such as the doctor-diagnosed asthma definition from the American Thoracic Society standardization project [18], or the British Medical Research Council (BMRC) definition [19]. However, using a dichotomous definition for such a highly heterogeneous and underdiagnosed disease as asthma, particularly when the doctor-diagnosed asthma definition is used, might lead to misclassification and biased estimates of the investigated associations. In this context, the asthma symptom score—a multi-categorical measure based on several asthma symptoms—has been proposed to study risk factors of asthma in longitudinal studies [20,21]. The asthma symptom score additionally allows for the detection of changes in asthma symptoms over time, reflecting either asthma incidence or remission, relapse or persistence of the disease among participants with asthma. Objective measures of variable expiratory airflow limitation (e.g., lung function with reversibility test, bronchial hyperresponsiveness) and atopic status (specific Immunoglobulin E (IgE), skin prick tests) are key features of asthma diagnosis in clinical practice. However, they are rarely available in epidemiological studies, particularly in the case of large cohort studies, and when they are, it is not clear which combination of objective measures should be used [6].

Most studies on asthma control are clinical studies and few epidemiological studies in large populations have assessed asthma control in a comprehensive manner. Several composite assessment instruments have been developed to measure asthma control. The childhood asthma control test (cACT) is a validated tool that has been widely used to assess asthma control in children aged 4 to 11 years old [22]. Regarding asthma control in adulthood, the asthma control test (ACT) and the asthma control questionnaire (ACQ), which are both derived using information on symptom frequency, rescue therapy use, sleep interference and activity limitation (the ACT covers the preceding month, whereas the ACQ covers the preceding week and additionally requires spirometry measures), have been proposed by experts as the most appropriate standardized composite scores [23].

### 1.2. Assessment of Nutritional Factors in Epidemiological Studies 

Regarding the assessment of **diet** in nutritional epidemiology, studies on specific foods or nutrients have traditionally been conducted. However, several conceptual and methodological limitations have been raised [24] as people do not consume isolated foods or nutrients, but meals consisting of a complex combination of foods, which themselves contain nutrients that possibly interact with each other and make it difficult to disentangle their isolated/joint effects. Another problem, when investigating the simultaneous effects of different foods, is that statistically significant associations can more easily occur by chance, thus raising the need to account for multiple testing. For these reasons, studying dietary patterns, which are data-driven methods, and dietary scores, which are based on prevailing hypotheses and guidance about the role of nutrients or foods in disease prevention, has been proposed in order to study the effects of overall diet, rather than the effects of specific foods or nutrients. Many dietary scores have been proposed; the most widely used in respiratory epidemiology are the Mediterranean diet score [25] and the international Alternate Healthy Eating Index 2010 (AHEI-2010) [26], which evaluate the effects of overall diet quality. Other dietary scores have been proposed to assess specific biological properties of diet, such as the dietary total antioxidant capacity (TAC) [27] and the dietary inflammatory index (DII) [28]. Another challenge in nutritional epidemiology is the method used to collect dietary data. Three methods have been the most extensively used: (1) the short-term 24-hour recall and (2) diet record methods, which both allow for greater specificity and accuracy [29]; and (3) the food-frequency questionnaire (FFQ) method, which decreases the error of day-to-day consumption and shows overall good reproducibility and validity despite introducing errors caused by averaging over long time intervals [30]. 

To assess **physical activity**, most epidemiological studies have primarily relied on self-reported questionnaires. Although no standardized approach exists, the self-completed international physical activity questionnaire (IPAQ)—which provides information on the time spent walking, in vigorous- and moderate-intensity activity and in sedentary activity, as well as additional information on household and yard work activities, occupational activity and self-powered transport (for the long version of the questionnaire)—has been validated previously in multiple international settings and population groups [31]. Using information from self-reported questionnaires, physical activity can be estimated by multiplying the metabolic equivalent of task (MET) cost of each activity by their frequency and duration, following the official IPAQ scoring protocol [32]. Nevertheless, physical activity is best evaluated objectively by accelerometers, which are wearable monitor devices that measure multiaxial accelerations of the body segment to which they are attached, thus enabling the estimation of the amount of sedentary and physical activity time [33]. Accelerometers offer a valid, objective alternative to self-reported questionnaires, which suffer from recall bias, measurement error and insufficient validity [34], although their use in population-based or longitudinal studies may be less feasible.

Body mass index (BMI in kilograms per square meter), with thresholds proposed by the World Health Organization or the U.S. Centers for Disease Control and Prevention to define underweight, overweight and obesity in adulthood and childhood [35,36], respectively, is widely used in epidemiological studies to evaluate **body composition**, because weight and height can be assessed quite accurately, even by self-reporting [37]. However, the use of BMI in epidemiological studies does not allow researchers to distinguish between fat mass and lean body mass [38]. For instance, two persons with the same BMI may have different proportions of fat free mass and fat mass, and a higher fat mass/lean mass ratio is likely to have more adverse metabolic effects. Indeed, a recent study has suggested that studying body compartments is more relevant when assessing the effects of obesity on lung function and growth in children [39]. Several methods have been developed to measure fat mass and lean body mass, including densitometry [40] or dual-energy X-ray absorptiometry (DEXA) [41], and more recently, bioelectrical impedance analysis (BIA) [42], which is more easily applicable to epidemiological studies, but may not be superior to BMI as a predictor of overall adiposity in a general population [43]. Several alternative ways have been proposed to measure adiposity in epidemiological studies, such as the calculation of waist and hip circumference (and their ratio), skinfold measurements [38], or the use of body silhouette pictograms [44].

## 2. The Role of Nutritional Factors in Asthma and Asthma Control: State of the Art

Nutritional factors evolve throughout life and may thus influence asthma and its control in different ways and by different mechanisms according to the window of exposure. It is thus crucial to make the distinction between the prenatal period, childhood and adulthood when comparing findings from the literature. The bulk of research investigating nutritional factors in asthma and asthma control has focused on a single nutritional factor, while considering the other nutritional factor(s) as potential confounders (when available). In the next section, we give a general overview (i.e., a non-exhaustive review) of the scientific literature on each nutritional factor and asthma and asthma control, distinguishing findings by windows of exposure (antenatal/childhood/adulthood) and by outcomes (asthma versus asthma control).

### 2.1. Diet and Asthma and Its Control

#### 2.1.1. Asthma

Evidence for a protective role of maternal intake of vitamins E and D, zinc, fruits, vegetables, omega-3 polyunsaturated fatty acids (PUFAs) or an overall healthy diet (assessed using the Mediterranean diet score or a negative DII score) during pregnancy has been found with regard to the risk of wheezing in the first years of life [45,46,47,48,49,50], but evidence for a lower risk of actual asthma, or even wheezing later in childhood, is lacking [45,46,47,48,51,52]. Recent studies have suggested a protective effect of maternal iron supplementation [53] and deleterious effects of high maternal free sugar, fructose and sugar-sweetened beverage (SSB) intake [54,55] and of a pro-inflammatory diet [56] during pregnancy on asthma development in mid- or late-childhood.

Many studies have investigated the role of diet during childhood, but very few studies were longitudinal. The association between diet and childhood asthma is moderate at best, with observational studies reporting ‘protective’ associations for fruits and vegetables and fish intake [45,57,58]. In recent years, several studies—including one longitudinal study—have suggested a deleterious effect of high fructose and SSB intake on asthma development [55]. Very few longitudinal studies have investigated the association between dietary patterns and childhood asthma, including one suggesting a deleterious effect of a Western diet (i.e., a high intake of refined grains, savory and snacks, sugar-containing beverages, meat, etc.), derived using principal component analysis (a data-driven approach), on wheeze incidence [59].

Few longitudinal studies have investigated the role of diet during adulthood. The association between diet and adult asthma is moderate at best, with observational studies reporting ‘protective’ associations for fruits and vegetables and vitamin E [57,60]. Recent studies using the asthma symptom score as a continuous definition of asthma have shown evidence for a role of diet at the nutrient level (a protective role of high fiber intake [61]), at the food level (a deleterious role of high processed meat intake [62,63]) and at the dietary score level (a protective role of a healthy diet [64,65]), whereas no evidence was found using a binary definition for asthma [66,67,68]. In recent years, several studies—including one longitudinal study—suggested a deleterious effect of SSB intake on asthma [69].

#### 2.1.2. Asthma Control

The Global Initiative for Asthma Guidelines encourages patients with asthma to consume a diet high in fruit and vegetables for its general health benefits [6]. But to date, the role of diet as a disease modifier remains underexplored. In children, a few studies reported that higher serum levels of 25(OH)D may be associated with a reduced risk of asthma exacerbations [70], whereas no evidence for a role of PUFAs on asthma control has been found so far in children or adults [60]. In adults, evidence was found for a deleterious effect of the ‘Western’ dietary pattern on asthma attack frequency [71] and asthma control [72], for a protective effect of a ‘nuts and wine’ pattern on asthma attack frequency [71] and for a protective effect of the “Prudent” pattern (i.e., characterized by a high intake of fruits, vegetables, whole grains, legumes, etc.) and healthy dietary scores on asthma control [65,72], whereas the evidence regarding the effect of the Mediterranean diet on asthma control was conflicting [73,74]. More recently, a pilot randomized controlled trial (RCT) reported better asthma control after a 6-month healthy eating intervention based on the Dietary Approaches to Stop Hypertension (DASH) score [75]. 

### 2.2. Physical Activity and Asthma and Its Control

#### 2.2.1. Asthma

Only a few longitudinal studies have been conducted on the role of physical activity in asthma development, suggesting overall a protective effect both in children and in adults, but studies are very heterogeneous in terms of the assessment of asthma and physical activity [76,77]. Moreover, reverse causation is a major limitation in the investigation of the role of physical activity in asthma development.

#### 2.2.2. Asthma Control

Despite national and international guidance to increase exercise, patients with asthma are less likely to engage in physical activity, partly due to the risk of exercise-induced bronchoconstriction (EIB). Although some intervention studies of exercise as a disease-modulating treatment have suggested clinical improvements for obese and non-obese patients with asthma, and exercise is encouraged in current treatment guidelines, there are no specific recommendations as to the intensity, frequency or duration of exercise exposure. This research is urgently needed so that more specific physical activity recommendations can be developed for children and adults with asthma [78,79,80].

### 2.3. Body Composition and Asthma and Its Control

#### 2.3.1. Asthma

Obesity is now an established risk factor for asthma [10,81]. Regarding windows of exposure, although several studies have suggested that a high BMI during childhood may predict a risk of developing asthma in adulthood [82], others have suggested that being overweight early in life may not have a long lasting effect on childhood asthma, if the child develops a normal weight later on [83]. Other studies concluded that a high BMI in adulthood, or an increase in body silhouette between menarche and adulthood, is related to the incidence of asthma later in life [84]. Although a low birth weight has been associated with childhood asthma [85], a recent meta-analysis points to the deleterious effect of both pre-pregnancy maternal obesity or overweight and high or low gestational weight gain on the childhood asthma risk in the offspring [86], supporting the early origins hypothesis for asthma. Further longitudinal studies accounting for the tracking of body composition from pregnancy to adulthood are thus needed [85]. Although the direction of the obesity-asthma association has been established for a long time (i.e., obesity preceding asthma) [87], a few recent studies have suggested that asthma may also precede childhood obesity [88,89]. A number of studies have then used Mendelian randomization (i.e., an approach using genetic variants to assess causal relationships, which can be regarded as a ‘natural’ RCT) to test the bi-directional association between obesity and asthma. Although these studies support the hypothesis that obesity is causally related to asthma, they did not find evidence supporting the causal role of asthma on obesity.

#### 2.3.2. Asthma Control

A review of 11 studies reported an overall positive association between obesity and worse asthma control in asthma patients [11]. The obesity–asthma control association has been further strengthened by consistent findings showing that weight reduction in obese patients with asthma, through bariatric surgery or not, was associated with improvement in multiple asthma-related outcomes, including asthma control [90].

In brief, published papers investigating nutritional factors in asthma and its control are heterogeneous in terms of the conceptual definition and assessment of both the outcomes and the exposures, and in terms of the window of exposure, which may explain why much of this literature remains inconclusive.

### 2.4. The Complex Interrelations between Nutritional Factors and Asthma and Its Control

Although each of the three nutritional factors has individually been associated with asthma or asthma control in longitudinal studies for different windows of exposure, their combined effects across the life course has not received a lot of attention. However, the investigation of nutritional factors as a whole (e.g., the Western lifestyle) is highly relevant in terms of understanding underlying mechanisms, as well as designing effective public health interventions.

Several shared mechanisms have been proposed to explain the role of each nutritional factor on asthma and its control, including oxidative stress and inflammation, and more recently, imbalance in the gut microbiome. Indeed there has been an exponential increase in the evidence—mainly from animal studies—linking the gut microbiota’s dysbiosis with diseases such as obesity and asthma [91] and with dietary [92] and exercise [93] interventions, and evidence of a lung-gut microbiome axis [94,95,96]. Other specific mechanisms have been suggested to explain the role of nutritional factors in asthma and its control, such as mechanical or hormonal mechanisms or via comorbidities, concerning obesity [97]; the modulatory effect of sigh rate on smooth muscle function and bronchoconstriction, concerning a sedentary lifestyle [98] and physical activity [99]; and epigenetic modification [100] or vitamin D pathways, concerning diet [101]. It is thus particularly relevant to identify and distinguish possible shared and specific mechanisms and pathways that may underlie the complex interrelations between diet, physical activity, body composition and asthma across the life course. For example, the effect of low physical activity or unhealthy diet on asthma could partly be mediated by the inflammatory effect of fat mass and adipokines (an indirect effect via body composition), in addition to a direct effect through other specific mechanisms (e.g., the deleterious effect of a sedentary lifestyle on bronchoconstriction or epigenetic modulation from the diet).

A few interventional studies in asthma have considered more than one nutritional factor [102]. Among them, one RCT conducted among 330 obese adults with uncontrolled asthma, targeting modest weight loss (i.e., of 7–10%) through a 12-month intervention based on healthy-eating counseling with calorie reductions (but no specific dietary pattern) and moderate-intensity physical activity did not report evidence for a significant overall benefit for asthma control (although weight loss of 10% or greater was associated with improved asthma control) [103]. In contrast, another RCT conducted among 125 non-obese patients with asthma, targeting a high protein, low glycemic index, anti-inflammatory diet, and high-intensity interval training on indoor spinning bikes over 8 weeks reported improved asthma control and asthma-related quality of life [104]. However, interventional studies usually have a relatively short follow-up that does not allow us to make any long-term inferences, and most dietary intervention studies either focus on the effect of a specific nutrient or target relatively strict calorie reductions, and thus do not allow for the examination of lifestyle interventions that are realistically achievable in terms of public health. Observational studies are thus crucially needed to overcome these limitations. To date, few observational studies have investigated the joint roles of diet, physical activity and/or body composition as determinants of asthma, either by simply considering each nutritional factor as an independent exposure [105,106,107,108,109,110,111,112], or by creating a combined score of “unhealthy behaviors” (e.g., unhealthy weight, high processed meat intake, unhealthy overall diet and smoking) [63,113], but most of them do not properly address the methodological challenges that are inherent to the investigation of the role of nutritional factors in asthma. For example, one of these studies, which was a large cross-sectional study conducted in children from eight Spanish cities, suggested independent protective effects of the Mediterranean score and exercise and a deleterious effect of obesity with regards to current asthma [105]. However, the possible independent effect of each risk factor was assessed using standard regression models, adjusting one nutritional factor for the other, leading to the issue of over-adjustment. Another study assessed the interrelations between obesity, physical fitness, sedentary time and asthma incidence among children using generalized equation analysis and a structural equation model [112], suggesting that low physical fitness levels and high screen time leads to central obesity, which leads to asthma development. Although the study was longitudinal (in terms of associations between nutritional factors and childhood-onset asthma), interrelations between physical fitness level, sedentary time and central obesity were analyzed in a cross-sectional manner, leading to the issue of time-dependent confounding.

#### 2.4.1. The Issue of Mediation in the Interrelations between Diet, Physical Activity, Body Composition and Asthma

One major methodological challenge in the investigation of nutritional factors in asthma comes from the fact that diet, physical activity and body composition are lifestyle factors that are closely interrelated (overweight and obesity reflect an imbalance between energy provision—i.e., intake of calories—and expenditure—i.e., physical activity), which makes it difficult to disentangle their separate effects on a health outcome. In particular, body composition might mediate potential effects of diet or physical activity on an obesity-related disease, such as asthma (Figure 1). Previous statistical and epidemiological research has shown that adjusting for a mediator (e.g., obesity) and a common cause (e.g., physical activity) of the mediator and the outcome, when assessing the causal association between an exposure (e.g., dietary habits) and an outcome (e.g., asthma), leads to biased results [114,115]. Despite the complex interrelationships within nutritional factors, obesity (or BMI) has usually been considered as a confounder in studies investigating the association between diet/physical activity and obesity-related diseases, raising the issue of potential over-adjustment.

Over the past 30 years, there have been important developments in causal theories, and novel approaches from the causal inference framework are now widely recommended to limit bias in epidemiological studies. In particular, the counterfactual approach (counterfactuals are defined as the outcome that would have been observed, had the exposure differed) in mediation analysis, has been proposed to tackle the issue of potential mediation by body composition in the effects of diet and physical activity on asthma [116,117]. These approaches enable researchers to distinguish and estimate the overall effect of the exposure on the disease (i.e., total effect), the effect passing through the mediator (i.e., indirect effect) and the effect unexplained by the mediator (i.e., direct effect) (Figure 1). Several epidemiological studies have used mediation analysis from the causal inference framework in the context of respiratory health [62,64,118,119,120,121,122], including studies on obesity and asthma [118], on obesity and lung function [119] and on physical activity and lung function [120]. One recent study was conducted to investigate potential mediation by obesity in the association between diet and healthy aging [123]. To our knowledge, only two studies were conducted in the context of nutritional factors and asthma, suggesting that BMI partly mediates the association between high cured meat intake and worsening asthma symptoms over time [62], but does not mediate the association between overall diet quality—assessed using the AHEI-2010—and improved asthma symptoms [64] (see Table 1).

#### 2.4.2. The Issue of Time-Dependent Confounding in the Interrelations between Nutritional Factors and Asthma

**Figure 2:**
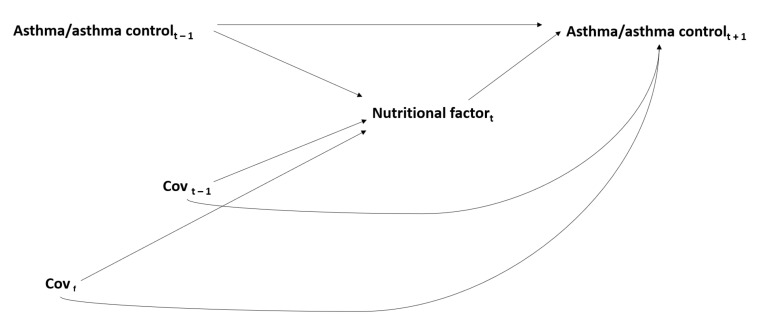
Issue of time-dependent confounding* in the interrelations between nutritional factors and asthma/asthma control. Cov_f_: time-fixed covariates; Cov_t − 1_: time-dependent covariates at time t − 1; * For simplicity, the interrelations between each nutritional factor are not represented in this figure (see Figure 1).

From a longitudinal perspective, interrelations between nutritional factors and asthma are time-dependent. In addition to the potential role of each nutritional factor at a given time t on asthma at a time t+1, asthma at time t − 1 may have modified nutritional factors at time t (e.g., asthma can lead to a decrease in physical activity), and each nutritional factor at time t − 1 may have modified another nutritional factor at time t (e.g., overweight/obesity can lead to modifying dietary and/or physical activity behaviors) (Figure 2). Thus, with the issue of the tracking of lifestyle habits and asthma/asthma control across the life course, and given the importance of windows of exposure in the investigation of the role of nutritional factors in asthma, the potential for reverse causation remains an issue, even in longitudinal studies where repeated data are available. Thus, there is an additional issue with time-dependent confounding that needs to be accounted for and, in this situation, standard methods of analysis may provide biased results [114,115].

In longitudinal epidemiological studies, time-dependent confounding can be addressed using novel approaches from the causal inference framework, such as marginal structural models (MSMs) [124]. These models, applicable only with repeated data, have been developed in the frame of the counterfactual approach to causality through inverse probability weighting [125]. This approach allows the estimation of causal effects in observational studies by mimicking a hypothetical randomized experiment via the creation of a pseudo-population in which exposed and non-exposed subjects are exchangeable within levels of the available confounders. The MSM approach has been rarely used in respiratory epidemiology [126,127,128,129,130]. Two studies were conducted to assess the time-dependent associations between physical activity and lung function [128,130] or physical activity and COPD [128]. MSMs have been used in the context of nutritional factors to study the joint effect of physical activity and body composition on functional limitation [131]. To our knowledge, only one study has been conducted in the context of the time-dependent associations between physical activity, BMI and asthma, suggesting an independent causal deleterious effect of overweight and obesity on current asthma, but no independent causal effect of physical activity on current asthma [129] (see Table 1).

The g-formula, which was first described in 1986 by Robins [132], is another counterfactual method that allows adjustment for time-dependent confounding. The g-formula is a generalization of standardization for time-dependent confounders and exposures, and can be used to consistently estimate the standardized risk of a health outcome under hypothetical interventions. The g-formula has been widely used to investigate the effect of potential exercise, dietary and/or weight loss interventions in relation with mortality [133], prostate cancer survival [134], periodontitis risk [135] and coronary heart disease (CHD) [136,137,138]. The g-formula approach has been rarely used in respiratory epidemiology [139,140,141]. To our knowledge, only one study has used the parametric g-formula in the context of nutritional factors and asthma—in this study, the authors assessed the 10-year risk of adult-onset asthma after hypothetical interventions on BMI and physical activity, and showed a significant reduction in asthma risk associated with weight loss intervention and a non-significant reduction associated with intervention on physical activity level [139] (see Table 1).

Recent developments, generalizing the g-formula to the mediation approach, have further enabled the investigation of interventional direct and indirect effects in the context of available time-dependent exposures and mediators [142]. This approach has already been used to investigate the mediating role of physical inactivity in the association between adult obesity and mid-life physical functioning [143].

## 3. The Association between Nutritional Factors and Asthma: An Epiphenomenon of the Complex Interrelations between Genetic, Environmental, Lifestyle and Social Determinants in Asthma

Aside from the complex interrelations between diet, physical activity, body composition and asthma, nutritional factors may also interact with genetic, environmental, lifestyle and social factors. Few studies have been published regarding this important issue despite the fact that asthma is considered to be caused by a complicated interplay of genetic, environmental, lifestyle and social factors. One way to strengthen causal inference is to demonstrate biologically plausible interactions. A few studies have investigated possible interactions between common antioxidant gene polymorphisms and antioxidant intake or physical activity affecting atopic and respiratory outcomes [144,145,146]. As obesity is now a well-known risk factor for asthma, trying to understand better how obesity might modify associations between oxidant exposures, such as air pollution, and asthma is particularly relevant [147]. Some studies have suggested that diet may act as an effect modifier and modulate the adverse effect of air pollution on asthma outcomes [148,149,150]. Investigating the potential interaction between exercise and air pollution, with the issue of whether or not increased exposure to air pollution during exercise outweighs the beneficial effects of physical activity on asthma is also critical in order to guide public health interventions [144,151]. Investigating interactions with smoking is particularly relevant given the complex association between smoking and body weight, and that smokers and non-smokers have different dietary habits and levels of physical activity [152]. Some studies have suggested that diet may act as an effect modifier and attenuate the deleterious effect of smoking on asthma [153], whereas others have suggested that a healthy diet may only be beneficial in never-smokers [64]. Investigating potential interactions between nutritional and social factors is also very relevant given the strong influence of social context on the development of dietary behaviors, lifestyle habits and health outcomes throughout life, and increasing research is focusing on “the exposome” concept in asthma [154,155].

## 4. Conclusions

The investigation of nutritional factors as a whole is highly relevant in the etiology of asthma and its control, both in terms of understanding their underlying mechanisms and in terms of guiding efficient multidimensional public health interventions. To date, few studies on the role of nutritional factors in asthma have properly addressed the methodological challenges posed by the complexity of their interrelations. Therefore, there is a crucial need for further studies investigating these interrelations and for more caution when addressing these conceptual and methodological challenges in the conduct of analysis and the comparison of results. By providing guidance on how to address these challenges, we hope this review will help to identify current research needs and guide future research.

## Figures and Tables

**Table 1:** Studies that used methods from the causal inference framework to investigate associations between nutritional factors and asthma.

Reference	Population	Design	Outcome	Exposures	Results	Comments
Li Z et al., *Thorax* 2017	971 adults from the French prospective EGEA case-control study (baseline: 2003–2007; follow-up: 2011–2013)	Mediation analysis in the counterfactual framework to estimate the direct effect of baseline cured meat intake on change in asthma symptom over follow-up, and the indirect effect mediated by BMI at baseline	Change in asthma symptom score (calculated at each time-point using information as sum of 5 respiratory asthma symptoms in the last 12 months) categorized as ‘stable/improved’ or ‘worsening’	Cured meat intake (<1, 1–3.9, ≥4 servings/week) estimated using information on average dietary intakes during the previous 12 months of ham, dried sausage and sausage consumption (from a 118-item semi-quantitative food-frequency questionnaire (FFQ) based on a French validated dietary questionnaire) BMI: calculated using measures of height and weight	Positive direct effect of cured meat intake on worsening asthma symptoms: multivariable odds ratio (OR) = 1.76 (95% CI: 1.01, 3.06) for ≥4 vs. <1 serving/week Positive indirect effect mediated by BMI: OR=1.07 (1.01, 1.14) for ≥4 vs. <1 serving/week, accounting for 14% of the total effect	Physical activity at baseline, expressed in metabolic equivalents (METS)/week, was considered as potential confounder and thus adjusted for in the models
Li Z et al., *Br J Nutr* 2017	969 adults from the French prospective EGEA case-control study (baseline: 2003–2007; follow-up: 2011–2013)	Mediation analysis in the counterfactual framework to estimate the direct effect of baseline AHEI score on change in asthma symptom over follow-up, and the indirect effect mediated by BMI at baseline	Change in asthma symptom score (calculated at each time-point using information as sum of 5 respiratory asthma symptoms in the last 12 months) categorized as ‘stable/improved’ or ‘worsening’	The AHEI-2010 dietary score (range 0-10 based on high intake of vegetables, fruits, whole grains, nuts and legumes, long-chain n-3 fatty acids and PUFA; moderate intake of alcohol; and low intake of sugar-sweetened drinks and fruit juice, red/ processed meat, trans-fat and Na), estimated using information from a 118-item semi-quantitative FFQ based on a French validated dietary questionnaire BMI: calculated using measures of height and weight	- Among never smokers: positive total effect (multivariable OR = 1.39 [1.07, 1.80] and positive direct effect (OR = 1.41 [1.09, 1.80] of the AHEI-2010 (per ten-point increment) on improved symptoms; no indirect effect mediated through BMI (OR= 0.99 [0.91, 1.07]). - Among former and current smokers: no statistically significant effect	Physical activity at baseline, expressed in metabolic equivalents (METS)/week, was considered as potential confounder and thus adjusted for in the models
Bédard A et al., *Am J Epidemiol* 2017	15,353 adult women from the Asthma-E3N case-control study (nested within the French E3N cohort) with data collected at least 4 times between 1997 and 2011	Marginal structural models (MSMs) considering three time periods: t − 1/t/t + 1 (1997/2000/2002, 2000/2002/2005, and 2002/2005/2011) with BMI and physical activity at time t, current asthma at time t + 1, and covariates at time t − 1 or baseline	Current asthma: asthma attacks and/or asthma treatment (inhaled bronchodilators or inhaled corticosteroids) in the last 12 months (self-report)	BMI: calculated using self-reported height and weight Physical activity: expressed in metabolic equivalent of task (MET)-hours per week using self-reported amount of time spent doing different activities. All MET-hours/week values were added and categorized in tertiles (low/moderate /high level of physical activity)	- Strong significant and positive dose–response relationship between BMI and current asthma: OR = 0.90 (0.79, 1.03), 1.29 (1.17, 1.42) and 1.87 (1.60, 2.18) for the BMI groups < 20.0, 25.0–29.9, and ≥30.0 respectively, versus the normal-weight group (BMI 20.0–24.9). - No association between physical activity and current asthma	Information on diet was only available once, and thus total daily energy intake (assessed using a validated FFQ) was considered as a time-fixed covariate in the MSMs
Garcia-Aymerich J et al., *Am J Epidemiol* 2014	76,470 asthma-free women from the Nurses’ Health Study who were followed between 1988 and 1998	g-formula analysis to assess the 10-year risk of adult–onset asthma after hypothetical interventions on BMI (i.e., reducing BMI by 5% every 2 years) or/and physical activity (i.e., engaging in at least 2.5 h per week of moderate-to-vigorous physical activity)	Adult-onset asthma: self-reported physician diagnosis of asthma plus the use of an asthma medication in the past 12 months	BMI: calculated using self-reported height and weight Physical activity: total time spent per week at moderate-to-vigorous physical activities (walking at ≥3 miles/hour, hiking outdoors, jogging, running, cycling, swimming, tennis and calisthenics/aerobics/aerobic dance/rowing machine)	Compared with no intervention, the population risk ratios were 0.96 (0.93, 0.99) under the BMI intervention, 0.96 (0.81, 1.10) under the physical activity intervention, and 0.92 (0.78, 1.06) under the joint intervention	Because of a large proportion of missing data, diet (assessed the dietary “prudent pattern” and “Western pattern”) was considered as a time-fixed covariate
